# Analysis of D-A locus of tRNA-linked short tandem repeats reveals transmission of *Entamoeba histolytica* and *E*. *dispar* among students in the Thai-Myanmar border region of northwest Thailand

**DOI:** 10.1371/journal.pntd.0009188

**Published:** 2021-02-18

**Authors:** Urassaya Pattanawong, Chaturong Putaporntip, Azumi Kakino, Naoko Yoshida, Seiki Kobayashi, Surasuk Yanmanee, Somchai Jongwutiwes, Hiroshi Tachibana

**Affiliations:** 1 Molecular Biology of Malaria and Opportunistic Parasites Research Unit, Department of Parasitology, Faculty of Medicine, Chulalongkorn University, Bangkok, Thailand; 2 Department of Parasitology, Tokai University School of Medicine, Isehara, Japan; 3 Department of Tropical Medicine and Parasitology, Faculty of Medicine, Juntendo University, Tokyo, Japan; 4 Department of Infectious Diseases, Keio University School of Medicine, Tokyo, Japan; National University of Singapore, SINGAPORE

## Abstract

Intestinal parasitic infections, including those caused by *Entamoeba* species, are a persistent problem in rural areas of Thailand. The aims of this study were to identify pathogenic *Entamoeba* species and to analyze their genotypic diversity. Stool samples were collected from 1,233 students of three schools located in the Thai-Myanmar border region of Tak Province, Thailand. The prevalence of *Entamoeba* infection was measured by polymerase chain reaction (PCR) using species-specific primers. Thirty-one (2.5%) positive cases were detected for *E*. *histolytica*, 55 (4.5%) for *E*. *dispar*, and 271 (22.0%) for *E*. *coli*. Positive samples for *E*. *histolytica* and *E*. *dispar* were exclusively obtained from a few school classes, whereas *E*. *coli* was detected in all grades. No infections caused by *E*. *moshkovskii*, *E*. *nuttalli*, *E*. *chattoni*, and *E*. *polecki* were detected in the students studied. The D-A locus of tRNA-linked short tandem repeats was analyzed in samples of *E*. *histolytica* (n = 13) and *E*. *dispar* (n = 47) to investigate their diversity and potential modes of transmission. Five genotypes of *E*. *histolytica* and 13 genotypes of *E*. *dispar* were identified. Sequences of the D-A were divergent, but several unique genotypes were significantly prevalent in limited classes, indicating that intra-classroom transmission has occurred. As it was unlikely that infection would have been limited within school classes if the mode of transmission of *E*. *histolytica* and *E*. *dispar* had been through the intake of contaminated drinking water or food, these results suggest a direct or indirect person-to-person transmission mode within school classes. Positive rates for three *Entamoeba* species were 2-fold higher in students who had siblings in the schools than in those without siblings, suggesting that transmission occurred even at home due to heavy contacts among siblings.

## Author summary

Transmissions in endemic areas of the pathogen *Entamoeba histolytica* and other non-pathogenic *Entamoeba* species such as *E*. *dispar* and *E*. *coli* are caused by ingestion of drinking water and foods contaminated with cysts of the parasites. Cases of *Entamoeba* infections among school-aged children have been reported in several countries. However, it has not been demonstrated that transmission of protozoa of the *Entamoeba* genus occurs within school facilities. In addition, genetic information on *E*. *histolytica* and other morphologically indistinguishable species, including *E*. *dispar* and *E*. *moshkovskii*, in Thailand remains scarce. In the present study, we demonstrated that *E*. *histolytica* and/or *E*. *dispar* are prevalent among school-aged children, but limited to few classes in three rural schools in the Thai-Myanmar border region of northwest Thailand. Although various genotypes of these *Entamoeba* species were identified, identical genotypes were significantly more prevalent in certain school classes and also among siblings, suggesting that transmission occurred within the classrooms and at home. The possibility of person-to-person transmission among these students via direct or indirect contact during daily activities in classrooms and home is proposed.

## Introduction

Intestinal parasitic infections are a persistent problem in rural areas of Thailand [1–5]. Chronic intestinal parasitic infections can cause malnutrition, anemia, growth retardation, and intellectual developmental delays in preschoolers and students. As such, cross-sectional studies on intestinal parasitic infections have been conducted in Thai children from various locations [6–9]. Microscopy studies also demonstrated the prevalence of *Entamoeba*. However, as *Entamoeba histolytica* is morphologically indistinguishable from species such as *E*. *dispar* and *E*. *moshkovskii*, the prevalence of each species in Thailand remains unknown. *E*. *histolytic*a is the pathological agent of amebiasis, which is associated with an estimated 50 million cases of colitis and liver abscesses that result in 55,000 deaths per year worldwide. *E*. *dispar* has mainly been isolated from cysts in fecal samples from asymptomatic carriers and is described as non-pathogenic. *E*. *moshkovskii* is primarily free-living, but human infections have also been reported in many countries [10–12].

Recent studies have reported polymorphisms for a serine-rich protein gene, chitinase gene, and tRNA-linked short tandem repeats (tRNA-STR) in *E*. *histolytica* and *E*. *dispar* [13–15]. The tRNA-STR polymorphism is highly divergent, and several reports have correlated specific genotypes with symptoms [16–18]. Genotyping is a useful tool that can be used to investigate the dynamics of infection in families, communities, and schools [19–21]. However, epidemiological studies to identify *Entamoeba* species and characterize their genotypic diversity have not been previously conducted in schools in Thailand.

Here, we report the prevalence of intestinal parasites, including protozoa and helminths, with a special focus on *Entamoeba*, among students from schools located in the Thai-Myanmar border region in northwest Thailand. Genotypic analyses of *E*. *histolytica* and *E*. *dispar* suggested a mode of person-to-person transmission in classrooms and in the home.

## Methods

### Ethics statement

This study was conducted in accordance with ethical protocols approved by the Institutional Review Board in Human Research, Faculty of Medicine, Chulalongkorn University, Thailand (IRB Nos. 236/54 and 246/61). Written informed consent was obtained from all participants or from their parents or guardians prior to stool sample collection.

### Study area and collection of samples

**Fig 1:**
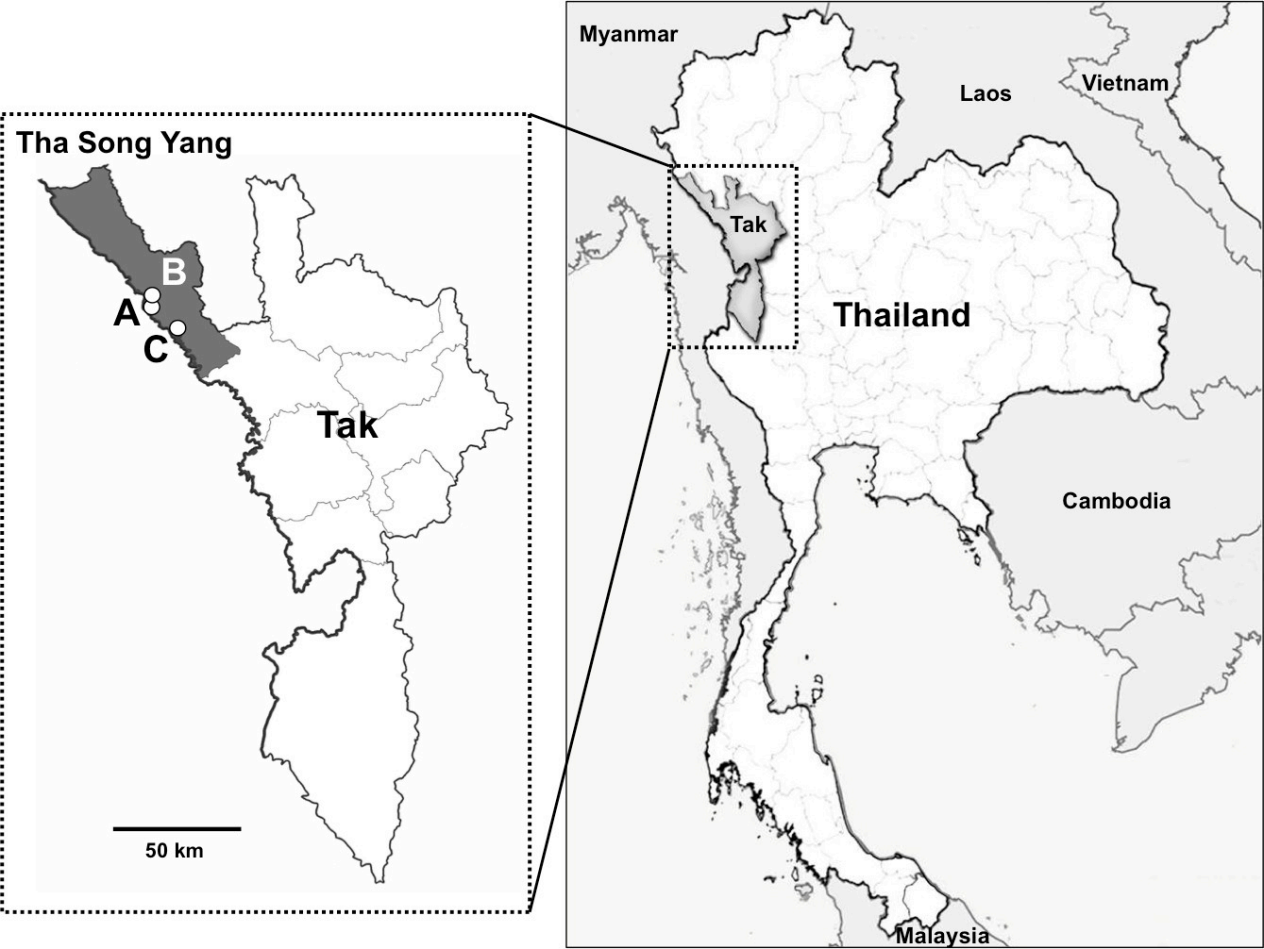
Map of the study area, Tha Song Yang District in Tak Province, Thailand. A, B, and C indicate the locations of the schools studied.

A cross-sectional study was conducted at three schools (A to C) located in the Thai-Myanmar border region in Tha Song Yang, the northwestern-most district of Tak Province, Thailand, in July 2018 (Fig 1). School A has a kindergarten (2 grades), primary school (6 grades), and secondary school (3 grades). School B is located on a hill, and is a branch of school A, comprising a small-scale kindergarten and primary school. School C is a secondary school (6 grades).

Clean, wide-mouthed screw-capped plastic containers and spatulas were distributed to the children (or their parents) with instructions for stool sample collection. All 1,788 students from three schools: school A (n = 1,144), school B (n = 82), and school C (n = 562), were requested to submit their stool samples. The next day, stool samples were collected, kept cool on ice, and transported to the laboratory at Chulalongkorn University, Bangkok. A total of 1,233 stool samples were obtained, accounting for 69% of total students from the three schools (70.6%, 53.7%, and 67.8% from schools A, B, and C, respectively). Main reasons for the students not providing their stool samples were no defecation on that morning or unwillingness to participate. Characteristics of students who participated in this study are summarized in Table 1. Herein, individual classrooms for kindergarten, primary, and secondary schools are referred to as ‘Kin-’, ‘Pri-,’ and ‘Sec-’, respectively, followed by classroom grades and specific rooms.

**Table 1:** General characteristics of students in the three schools.

Characteristics	School A (n) n = 808	School B (n) n = 44	School C (n) n = 381	Total (n) n = 1,233
**Educational stage (n)**				
*** *Kindergarten**	121	2	-	123
*** *Primary school**	448	42	-	490
*** *Secondary school**	239	-	381	620
**Sex (n)**				
*** *Male**	370	18	137	525
*** *Female**	438	26	244	708
**Age (years)**^**a**^				
*** *Range**	4 to 19	4 to 12	11 to 24	4 to 24
*** *Average**	10.9	9.8	15.4	12.2

^a^A few Hill-tribe students started school significantly later than other Thai children.

### Stool examination and culture

Stool samples were examined by light microscopy with iodine staining. Samples that contained *Entamoeba* quadrinucleate cysts were filtered through a Kim wipe, pelleted by centrifugation, and washed several times with distilled water. The samples were then cultured in Robinson’s medium at 37°C. Grown trophozoites were treated with a cocktail of antibiotics and were then cultured monoxenically with *Crithidia fasciculata* in YIMDHA-S medium supplemented with 15% adult bovine serum at 37°C [22]. Finally, some of the isolates were cultured axenically in the medium.

### Extraction of DNA and polymerase chain reaction (PCR) analysis

Genomic DNA was isolated from the stool samples using a QIAamp Fast DNA Stool Mini Kit (Qiagen). Genomic DNA from cultured trophozoites was isolated using a DNeasy Blood and Tissue Kit (Qiagen). PCR amplification of the partial *18S* rRNA genes of *E*. *histolytica*, *E*. *dispar*, *E*. *nuttalli*, *E*. *coli*, and *E*. *chattoni* was performed using primers specific for each species [23–25]. PCR amplification of the *18S* gene from *E*. *moshkovskii* was performed following the same condition described in a previous study but using newly designed primers [26]. Sequences of primers and annealing temperatures used are shown in Table 2. Genomic DNA isolated from cultured trophozoites of *E*. *histolytica* HM-1:IMSS, *E*. *dispar* SAW1734RclAR, *E*. *moshkovskii* Laredo, and *E*. *nuttalli* P19-061405 was used as a positive control. For *E*. *chattoni* and *E*. *polecki*, genomic DNA extracted from cysts in fecal samples of macaques and pigs was used as the positive control, respectively. The D-A locus of tRNA-STR was also amplified using common primers for *E*. *histolytica* and *E*. *dispar* [27].

**Table 2:** List of primers used for PCR analysis.

Primer name	Sequence (5′ to 3′)	Size of amplicon (bp)	Annealing temperature (°C)	References
Ehist-18S-S	GTTTTATACATTTTGAAGACTTTATG	434	60	[23]
Ehist-18S-AS	CAGATCTAGAAACAATGCTTCTCT			[23]
Edisp-18S-S	ATTTTATACATTTTGAAGACTTTACATT	458	60	[23]
Edisp-18S-AS	GAACAAGGTAGTATTGATATACTTG			[23]
Enut-18S-S	TTTTATACATTTTGAAGACTTTGCATA	452	60	[23]
Enut-18S-AS	AAGGTAATATTGATATACTCAGATTA			[23]
Ecoli-18S-S	GAATGTCAAAGCTAATACTTGACG	160	60	[24]
Ecoli-18S-AS	GATTTCTACAATTCTCTTGGCATA			[24]
Echattoni1	AGGATTTGTTTTATAACAAGTTC	215	55	[25]
Echattoni2	TAAATAACCTTTCTCCTTTTTCTATC			[25]
Epolecki1	TCGATATTTATATTGATTCAAATG	201	55	[25]
Epolecki2	CCTTTCTCCTTTTTTTAT ATTAG			[25]
Emoshkov3	TGACGACAAATAACTCTCGAGG	200	60	Present study
Emoshkov4	GCCTTCAAAATGATTAAAACCACC			Present study
D-A5 (EhR1)	CTGGTTAGTATCTTCGCCTGT	364–540^a^	58	[27]
D-A3 (EhR2)	GCTACACCCCCATTAACAAT			[27]

^a^Size range obtained in this study.

### Sequencing

PCR products were purified with either a QIAquick PCR purification kit (Qiagen) or QIAquick Gel Extraction Kit (Qiagen), and were sequenced using a BigDye Terminator v3.1 cycle sequencing kit (Applied Biosystems, Carlsbad, CA, USA) with an Applied Biosystems 3500 Genetic Analyzer (Applied Biosystems).

### Data analysis

Categorical data were computed as odds ratio (OR) with the 95% confidence interval (CI), and were compared between groups with the Chi-square test, with a difference considered to be statistically significant at *p* < 0.05. Data analysis was performed using Prism ver. 6.

## Results

### Prevalence of intestinal parasites observed by microscopy

The prevalence of each parasite was measured by microscopy, and is summarized in Table 3. Of the 1,233 students sampled, 393 (31.9%) were infected with at least one intestinal parasite. However, the prevalence varied significantly between schools. Only one student from school B (2.3%) was negative for intestinal parasites. In contrast, most of the students from schools A (69.6%) and C (72.7%) were negative. The most prevalent protozoon identified was *Blastocystis hominis* (10.5%), followed by *Endolimax nana* (10%), *E*. *coli* (9%), and *G*. *intestinalis* (4.9%). *E*. *histolytica*-like species was observed in 46 (3.7%) samples. *Ascaris lumbricoides* (5.1%) and *Trichuris trichiura* (1.5%) were the most prevalent helminths identified. Parasites that are transmitted through contaminated soil were highly prevalent in school B, with the highest prevalence observed for *A*. *lumbricoides* (61.4%) and *T*. *trichiura* (25%). In contrast, *A*. *lumbricoides* (0.8%) had a low prevalence and *T*. *trichiura* was not detected at school C.

**Table 3:** Prevalence of parasites observed by direct microscopy of simple fecal smears from students in Ta Song Yang District, Tak Province, Northwest Thailand.

Species	No. of positives (%)			
	School A n = 808	School B n = 44	School C n = 381	Total n = 1,233
**Protozoa**				
***Entamoeba histolytica-*like spp.**	28 (3.5)	7 (15.9)	11 (2.9)	46 (3.7)
***Entamoeba coli***	78 (9.6)	10 (22.7)	23 (6.0)	111 (9.0)
***Entamoeba hartmanni***	18 (2.2)	10 (22.7)	10 (2.6)	38 (3.1)
***Endolimax nana***	81 (10.0)	10 (22.7)	32 (8.4)	123 (10.0)
***Iodamoeba buetschlii***	6 (0.7)	8 (18.2)	4 (1.0)	18 (1.5)
***Giardia intestinalis***	49 (6.1)	3 (6.8)	8 (2.1)	60 (4.9)
***Chilomastix mesnili***	3 (0.4)	3 (6.8)	0 (0)	6 (0.5)
***Blastocystis hominis***	62 (7.7)	17 (38.6)	51 (13.4)	130 (10.5)
**Helminths**				
***Ascaris lumbricoides***	33 (4.1)	27 (61.4)	3 (0.8)	63 (5.1)
***Trichuris trichiura***	7 (0.9)	11 (25.0)	0 (0)	18 (1.5)
**Hookworm**	0 (0)	0 (0)	2 (0.5)	2 (0.2)
***Strongyloides stercoralis***	0 (0)	0 (0)	1 (0.3)	1 (0.1)
***Taenia* spp.**	1 (0.1)	0 (0)	2 (0.5)	3 (0.2)
**No. of negatives (%)**	562 (69.6)	1 (2.3)	277 (72.7)	840 (68.1)

### Prevalence of *Entamoeba* species detected by PCR

PCR was performed to increase the sensitivity of detection and to distinguish between *E*. *histolytica* and morphologically similar species. Thirty-one (2.5%) of the samples were positive for *E*. *histolytica*, 55 (4.5%) were positive for *E*. *dispar*, and 271 (22.0%) were positive for *E*. *coli* (Table 4). The prevalence of each species was the highest in school B and was the lowest in school C. The prevalence of *E*. *histolytica* and *E*. *dispar* was 2.5% and 4.8% in school A, 6.8% and 20.5% in school B, and 2.1% and 1.8% in school C, respectively. The prevalence of *E*. *dispar* significantly differed among the three schools (*p* < 0.0001 for school A vs B and B vs C; *p* = 0.0126 for school A vs C), whereas no significant difference was found among schools for the prevalence of *E*. *histolytica* (S1 Table). *E*. *histolytica* and *E*. *dispar* mixed infection was observed in only a single sample from school A. The positive rates for *E*. *coli* in schools A, B, and C were 23.4%, 72.7%, and 13.1%, respectively (*p* < 0.0001 among the three schools). Mixed infections of *E*. *histolytica* and *E*. *coli*, and of *E*. *dispar* and *E*. *coli* were observed in 2.6% and 7.4% of samples, respectively. No infections caused by *E*. *moshkovskii*, *E*. *nuttalli*, *E*. *chattoni*, or *E*. *polecki* were detected in all schools studied.

**Table 4:** Prevalence of *Entamoeba* infection detected by PCR analysis.

Species	No. of positives (%)			
	School A n = 808	School B n = 44	School C n = 381	Total n = 1,233
***Entamoeba histolytica***	20 (2.5)	3 (6.8)	8 (2.1)	31 (2.5)
***Entamoeba dispar***	39 (4.8)	9 (20.5)	7 (1.8)	55 (4.5)
***Entamoeba coli***	189 (23.4)	32 (72.7)	50 (13.1)	271 (22.0)
***Entamoeba moshkovskii***	0 (0)	0 (0)	0 (0)	0 (0)
***Entamoeba nuttalli***	0 (0)	0 (0)	0 (0)	0 (0)
***Entamoeba polecki* ST1**	0 (0)	0 (0)	0 (0)	0 (0)
***Entamoeba chattoni* (*E*. *polecki* S2)**	0 (0)	0 (0)	0 (0)	0 (0)

### Distribution of students positive for *Entamoeba* in each grade and class

**Fig 2:**
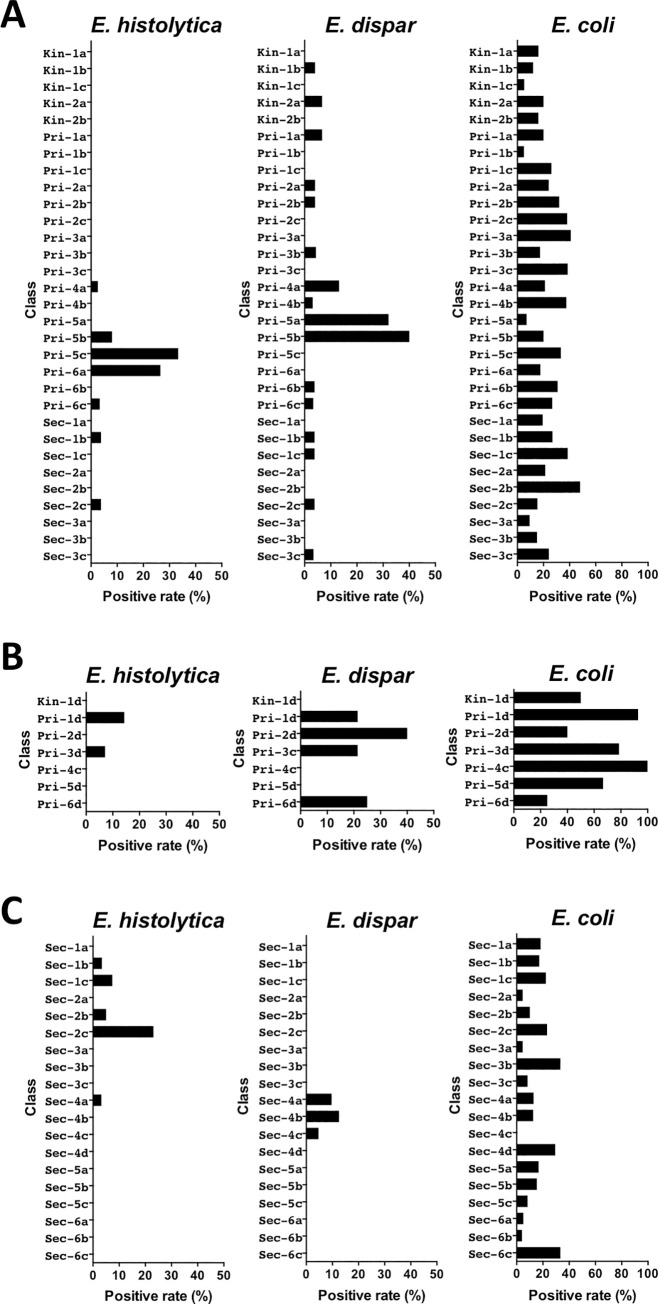
Distribution of students positive for *Entamoeba* in each class from schools A, B, and C.

The distribution of students in school A who were positive for *Entamoeba* is represented in Table 5. The 20 students who were positive for *E*. *histolytica* were exclusively from grade 4 of primary school through grade 2 of secondary school, with the greatest number of positives found in grades 5 (n = 7) and 6 (n = 10) of primary school. However, the distribution of students who were positive for *E*. *histolytica* was different among classes in grades 5 and 6 (Fig 2A). Of students who were positive, 5 (33.3%) were from class Pri-5c, 2 (8%) from Pri-5b, 9 (26.5%) from Pri-6a, and 1 (3.3%) from Pri-6c. No students from classes Pri-5a or Pri-6b were positive, whereas *E*. *dispar* was detected in all grades. However, the majority of students who were positive were from grades 4 and 5 (6 and 19 of 39, respectively) of primary school. Nine (32.1%) students from class Pri-5a and 10 (40%) students from Pri-5b were positive, but there was no positive result for *E*. *dispar* detected among students from Pri-5c. In contrast, *E*. *coli* was detected in all grades with a prevalence ranging from 13.6% to 32.4%. *E*. *coli* was also detected in all classes, with a prevalence varying from 5% to 48%.

**Table 5:** Distribution of *Entamoeba* positives in each grade for school A.

Educational stage	Grade	No. of students	No. of positives (%)		
			*E*. *histolytica*	*E*. *dispar*	*E*. *coli*
**Kindergarten**	1	66	0 **(**0**)**	1 **(**1.5**)**	9 **(**13.6)
	2	55	0 **(**0**)**	2 **(**3.6**)**	10 **(**18.2**)**
**Primary school**	1	75	0 **(**0**)**	2 **(**2.7**)**	13 **(**17.3**)**
	2	74	0 **(**0**)**	2 **(**2.7**)**	22 **(**29.7**)**
	3	71	0 **(**0**)**	1 **(**1.4**)**	23 **(**32.4**)**
	4	70	1 **(**1.4**)**	6 **(**8.6**)**	20 **(**28.6**)**
	5	68	7 **(**10.3**)**	19 **(**27.9**)**	12 **(**17.6**)**
	6	90	10 **(**11.1**)**	2 **(**2.2**)**	22 **(**24.4**)**
**Secondary school**	1	83	1 **(**1.2**)**	2 **(**2.4**)**	23 **(**27.7**)**
	2	77	1 **(**1.3**)**	1 **(**1.3**)**	22 **(**28.6**)**
	3	79	0 **(**0**)**	1 **(**1.3**)**	13 **(**16.5**)**
**Total**		808	20 **(**2.5**)**	39 **(**4.8**)**	189 **(**23.4**)**

In school B, we found that students who were positive for *E*. *histolytica* were exclusively from grades 1 and 3 of primary school (Table 6 and Fig 2B). Similarly, 8 of the 9 students who were positive for *E*. *dispar* were also from grades 1 to 3. However, students who were positive for *E*. *coli* were distributed across all grades, with prevalence varying from 25% to 100%.

**Table 6:** Distribution of *Entamoeba* positives in each grade for school B.

Educational stage	Grade	No. of students	No. of positives (%)		
			*E*. *histolytica*	*E*. *dispar*	*E*. *coli*
**Kindergarten**	1	2	0 (0)	0 (0)	1 (50.0)
**Primary school**	1	14	2 (14.3)	3 (21.4)	13 (92.9)
	2	5	0 (0)	2 (40.0)	2 (40.0)
	3	14	1 (7.1)	3 (21.4)	11 (78.6)
	4	2	0 (0)	0 (0)	2 (100.0)
	5	3	0 (0)	0 (0)	2 (66.7)
	6	4	0 (0)	1 (25.0)	1 (25.0)
**Total**		44	3 (6.8)	9 (20.5)	32 (72.7)

In school C, 7 of 8 students who were positive for *E*. *histolytica* were from grades 1 and 2 of secondary school (Table 7). One (3.4%) student from class Sec-1b, 2 (7.4%) from Sec-1c, 3 (23.1%) from Sec-2c, and 1 (5%) from Sec-2b were positive. No student in classes Sec-1a and Sec-2a was positive (Fig 2C). All 7 students who were positive for *E*. *dispar* were from grade 4 of secondary school. Three (9.7%) students from class Sec-4a, 3 (12.5%) from Sec-4b, and 1 (4.7%) from Sec-4c were positive, but no positives were detected in Sec-4d. In contrast, *E*. *coli* was detected in all grades, with the prevalence varying from 8.8% to 19.2%. Only class Sec-4c had no cases of *E*. *coli* infections; in other classes, the prevalence ranged from 4% to 33.3%.

**Table 7:** Distribution of *Entamoeba* positives in each grade for school C.

Educational stage	Grade	No. of students	No. of positives (%)		
			*E*. *histolytica*	*E*. *dispar*	*E*. *coli*
**Secondary school**	1	78	3 (3.8)	0 (0)	15 (19.2)
	2	55	4 (7.3)	0 (0)	6 (10.9)
	3	40	0 (0)	0 (0)	4 (9.8)
	4	93	1 (1.1)	7 (7.5)	12 (12.9)
	5	56	0 (0)	0 (0)	8 (14.3)
	6	59	0 (0)	0 (0)	5 (8.8)
**Total**		381	8 (2.1)	7 (1.8)	50 (13.1)

### Isolation of *E*. *histolytica* and *E*. *dispar*

Successful *in vitro* culture of samples containing quadrinucleate *Entamoeba* cysts in Robinson’s medium was achieved for 14 isolates. Of these, PCR identified *E*. *histolytica* in 5 isolates (school A, n = 2; school B, n = 3), while 9 isolates belonged to *E*. *dispar* (school A, n = 7; school B, n = 1; school C, n = 1). Genotypic analysis was subsequently performed. Three *E*. *histolytica* isolates (school A, n = 1; school B, n = 2) were established as axenic strains in YIMDHA-S medium.

### Genotypic analysis of *E*. *histolytica*

**Fig 3:**
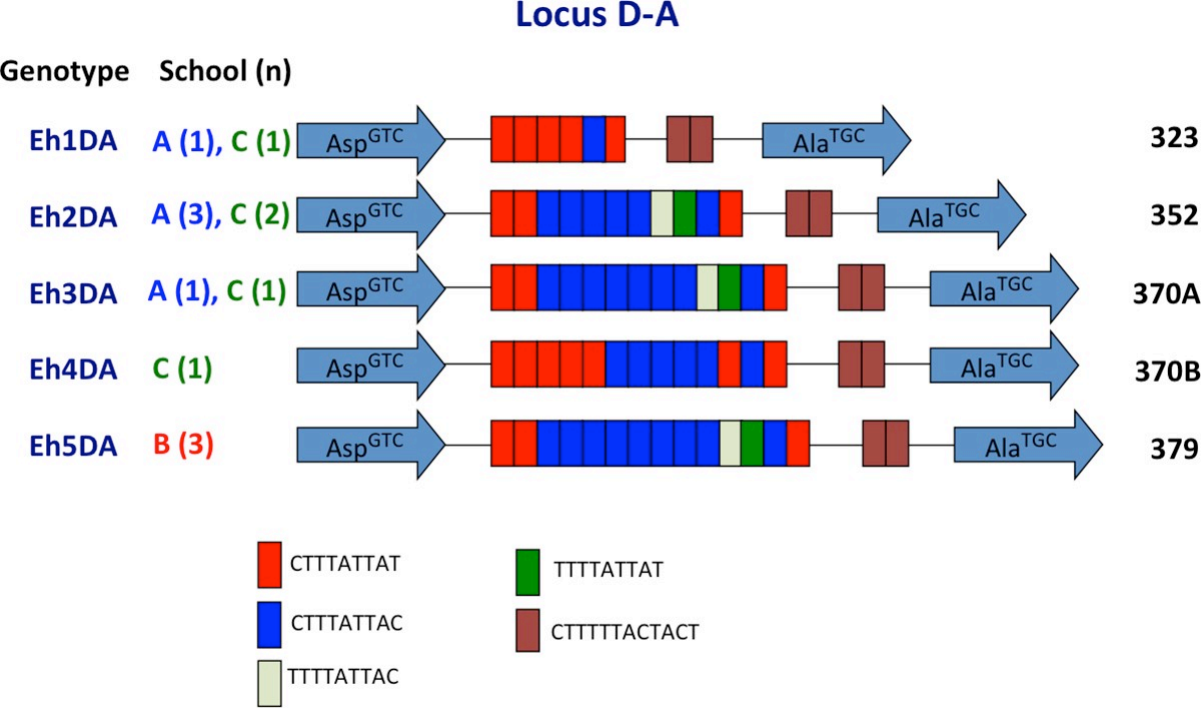
Schematic representation of tRNA-linked STR fragments at the D-A locus in *E*. *histolytica*. Numbers in parentheses indicate the number of samples with each genotype from each school. Numbers on the far right show the length of fragments excluding the primer sequences.

Sequencing was used to identify polymorphisms of tRNA-STR at the D-A locus. Sequences were successfully obtained for 13 (including 5 cultures) of 31 *E*. *histolytica* samples, and 47 (including 9 cultures) of 55 *E*. *dispar* samples. Five *E*. *histolytica* genotypes were identified (Fig 3). Three genotypes were identified in school A, 1 in school B, and 4 in school C. Of the 5 genotypes, 3 were common to schools A and C, and 2 were found exclusively in school B or school C (Fig 3). The most prevalent genotype (Eh2DA) was observed in 5 samples. The exclusive prevalence of genotype Eh5DA in school B was significant (*p* = 0.0047 in school A vs B and B vs C) (S2 Table). The prevalence in B-Pri-1d was also significnatly higher than the total prevalence from the other classes of the three schools (*p* = 0.0050).

### Genotypic analysis of *E*. *dispar*

**Fig 4:**
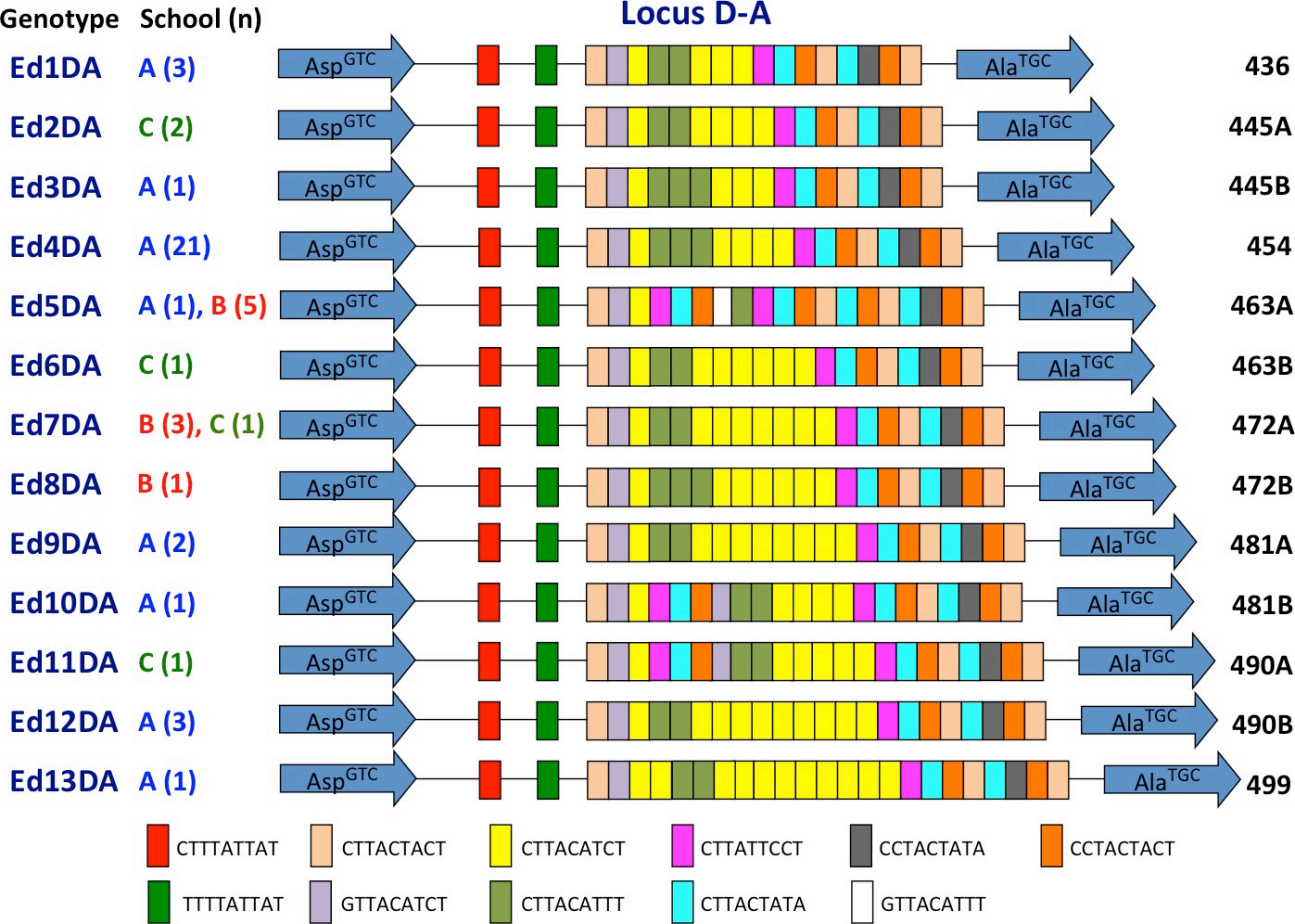
Schematic representation of tRNA-linked STR fragments at the D-A locus in *E*. *dispar*. Numbers in parentheses indicate the number of samples with each genotype from each school. Numbers on the far right show the length of fragments excluding the primer sequences.

**Fig 5:**
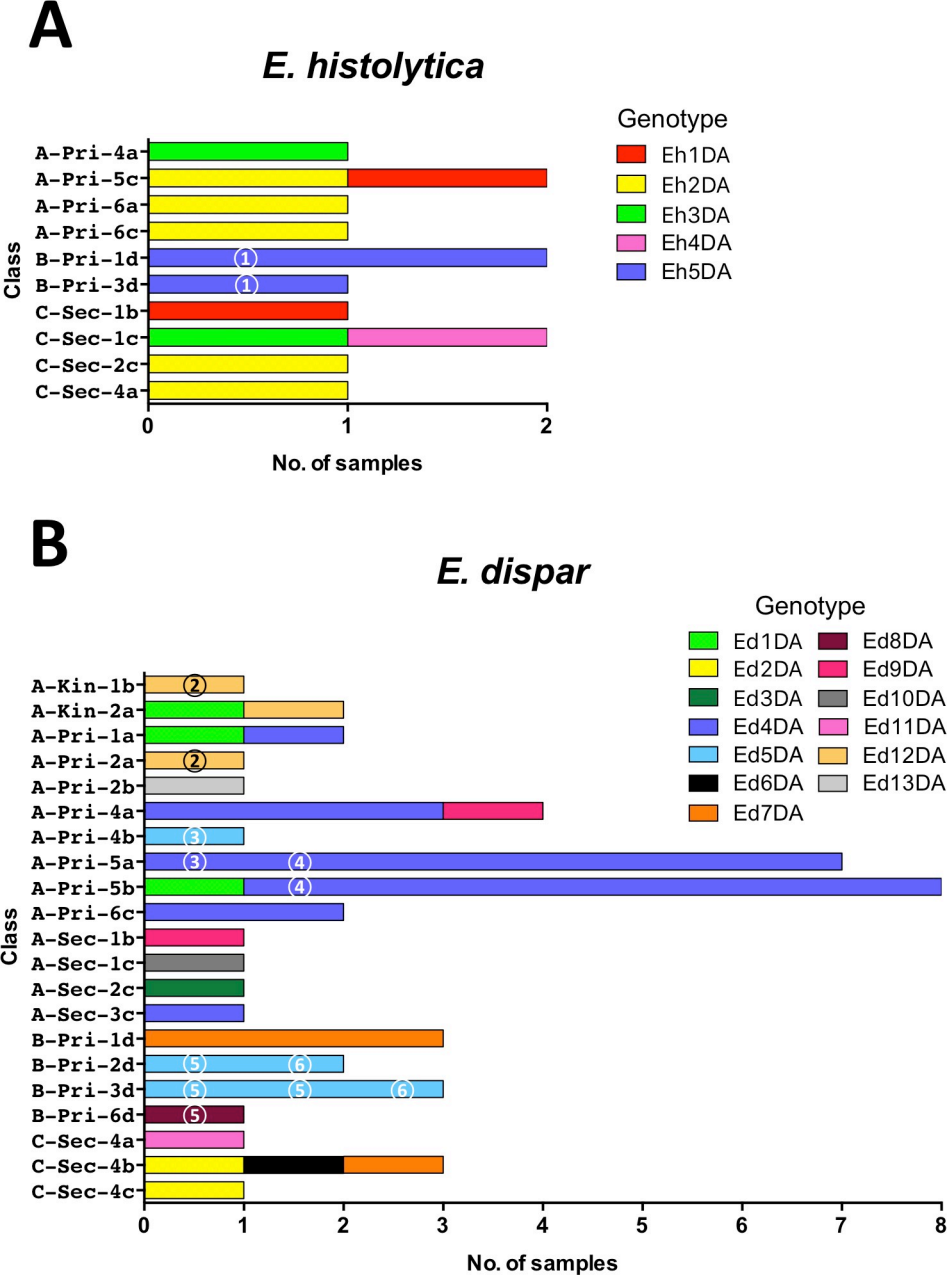
**Distribution of genotypes of tRNA-linked STR fragments at the D-A locus from *E*. *histolytica* (A) and *E*. *dispar* (B) in classes from three schools.** Different colors represent different genotypes. Identical numbers within circles represent siblings.

Thirteen *E*. *dispar* genotypes were identified in 47 samples, suggesting that it had relatively greater diversity (Fig 4). Eight genotypes were identified in school A, 3 in school B, and 4 in school C. Only two genotypes were common to different schools, and the 11 other genotypes were unique to each school. The most prevalent genotype, Ed4DA, was observed in 21 samples from school A, including all 7 positive samples from class Pri-5a and 7 of 8 samples from Pri-5b (Fig 5). The prevalence in Pri-5a was significantly higher than that in the remaining classes of school A (*p* = 0.0242) (S3 Table). The prevalence of Ed4DA in grade 5 (Pri-5a and Pri-5b) was also significantly higher than that in the remaining classes of primary school (*p* = 0.0204). These results indicated that transmission of *Entamoeba* occurred within classes and grades. Genotype Ed4DA was also prevalent in grades 4 (class Pri-4a) and 6 (class Pri-6c), whereas genotype Ed12DA was found exclusively in the lower grades of school A, including classes Kin-1b, Kin-2a, and Pri-2a. In contrast, the genotypes Ed3DA and Ed10DA were found exclusively in school A’s secondary school. In school B, genotype Ed7DA was prevalent in grade 1 (class Pri-1d), whereas genotype Ed5DA was prevalent in grades 2 (class Pri-2d) and 3 (class Pri-3d). The prevalence of Ed7DA in Pri-1d was significantly higher than that in the remaining classes of school B (*p* = 0.0027), indicating that intra-class transmission occurred (S4 Table). The prevalence of Ed5DA in Pri-3d and Pri-2d was also significantly higher than that in other classes of school B (*p* = 0.0027) (S5 Table). In class Sec-4b of school C, the 3 *E*. *dispar* samples had different genotypes: Ed2DA, Ed6DA, and Ed7DA.

### *Entamoeba* prevalence and genotypes in siblings

Of the 1,233 students included in this study, 400 had siblings in the same schools. The positive rates for three *Entamoeba* species among students who had siblings were about 2-fold higher than those among students who did not have siblings in their school (*p* = 0.0028 for *E*. *dispar* and *p* < 0.0001 for *E*. *coli*) (Table 8). Although the difference in *E*. *histolytica* infection prevalence was not statistically meaningful (*p* = 0.055), it almost reached a significant level. To study the transmission of *Entamoeba* within families, genotyping was used to identify siblings who were positive (Fig 5). Two pairs of siblings were among the 31 *E*. *histolytica*-positive children. The Eh5DA genotype of *E*. *histolytica* was shared with a sibling (circled 1) from school B, whereas genotypes from the other siblings from school A could not be analyzed. Moreover, siblings from six families were among the 55 *E*. *dispar*-positive children. Genotypes for *E*. *dispar* were compared in siblings from 5 families. In school A, siblings from 2 families (circles 2 and 4) had identical genotypes (Ed12DA and Ed4DA), but one pair of siblings (circle 3) had different genotypes (Ed4DA and Ed5DA). Genotypes from the remaining siblings from school A could not be analyzed. In school B, a pair of siblings (circle 6) had an identical genotype (Ed5DA), but a set of four siblings (circle 5) had different genotypes, with Ed8DA found in 1 sibling and Ed5DA in the others. No siblings from school C were found to be positive for *E*. *histolytica* and *E*. *dispar*.

**Table 8:** Comparison of *Entamoeba* infection prevalence between students with and without siblings in the school.

	No. of students	No. of positives (%)		
		*E*. *histolytica*	*E*. *dispar*	*E*. *coli*
**Students with siblings in the school**	400	15 (3.8)	28 (7.0)	137 (34.3)
**Students without siblings in the school**	833	16 (1.9)	27 (3.2)	134 (16.1)
**Chi-square**		3.689	8.959	51.991
** *p* value**		0.055	0.0028	<0.0001
**Odds ratio (95%CI)**		1.99 (0.97–4.07)	2.25 (1.31–3.87)	2.72 (2.06–3.59)
**Total**	1,233	31 (2.5)	55 (4.5)	271 (22.0)

## Discussion

This study revealed that the prevalence of intestinal parasites differed significantly among three schools located in the same district. School B, which is rural and small, had the highest prevalence, especially for nematodes transmitted through soil, such as *A*. *lumbricoides* and *T*. *trichiura*. In Thailand, a high prevalence of intestinal parasitic infections such as those caused by nematodes has been reported in children of the Karen Hill tribe and in immigrants from Myanmar [2,28,29]. We observed a much higher prevalence of *A*. *lumbricoides* in school B compared to that previously reported in Thailand [1–3]. Students who attended school B lived in close proximity to the school. Most of them lacked household latrines and drank untreated water from streams running through their villages. Therefore, the main reason for the high prevalence of parasitic infections in school B would be the poor hygiene. Further studies will be needed to investigate if the prevalence of this parasite is also relatively high within these students’ families.

The prevalence of *E*. *histolytica*/*E*. *dispar* and *E*. *coli* as detected by PCR was 6.9% and 22%, respectively, with a single mixed infection of *E*. *histolytica* and *E*. *dispar* detected. This was about twice as high as the prevalence observed by microscopy. Although concentration techniques may increase detection by microscopy, some studies have reported no difference for protozoa [3,29,30]. We showed that *E*. *dispar*, but not *E*. *histolytica*, was prevalent in the studied populations. Previous studies have reported that *E*. *dispar* was more prevalent than *E*. *histolytica* in hospitals in Bangkok [31,32] and that *E*. *histolytica* was prevalent in the Thai-Myanmar border region, such as in Phang-Nga Province [33].

Our main finding was that *E*. *histolytica* and *E*. *dispar* infections were distributed in a limited number of classes. In school A’s grade 5 of primary school, the number of samples positive for *E*. *histolytica* was 0 (0%) in class Pri-5a, 2 (8%) in Pri-5b, and 5 (33.3%) in Pri-5c. In the same classes, the number of samples positive for *E*. *dispar* was 9 (32.1%), 10 (40%), and 0 (0%), respectively, suggesting that the transmission occurred within classes. This also suggested that transmission of these two species must have occurred independently, even if both species shared the same mode of transmission. It has previously been reported that the D-A locus of tRNA-STR is highly variable and is thus useful for *Entamoeba* fingerprinting [15,19,21,34–36]. The present study showed that the prevalence of genotypes such as Ed4DA and Ed7DA was significantly higher in several classes, indicating that transmission occurred within the classroom. To our knowledge, this is the first report revealing the transmission of *Entamoeba* species within the classroom setting.

Concerning the high prevalence of Ed4DA in both Pri-5a and Pri-5b, in addition to intra-class transmission, the possibility that inter-class transmission between these two classes occurred could not be ruled out. However, it is difficult to prove whether transmission between these two classes occurred once (subsequent intra-class transmission), a few times, frequently, or did not occur at all (independent intra-class transmission) because of the cross-sectional nature of the present study. It is also reasonable to consider that contact between school children is longer and more extensive in the same classroom than in different classrooms. In fact, there was no *E*. *dispar-*positive case in A-Pri5c, despite being part of the same grade. Therefore, it is also reasonable that the incidence of intra-classroom transmission is higher than that of inter-classroom transmission. As different genotypes were found in secondary schools, this also suggests that children in primary schools have more person-to-person contact than older students. This study also provides evidence about the great genetic diversity of *E*. *dispar*, and demonstrates that the mobility of the parasite is restricted to relatively small areas where transmission is maintained [19–21].

In general, the mode of transmission for *Entamoeba* is through ingestion of contaminated drinking water or food [37–42]. However, the supplied drinking water at school A was filtered, and for lunch, children either bought food that was sold at the school or were provided with side dishes that were cooked in a central kitchen at the school. If water or food was the source of infection, it is unlikely that infections would be limited to specific classes. Therefore, the most likely route of transmission would be through direct or indirect person-to-person contact during daily activities in classes and in grades. It is also improbable that *Entamoeba* was transmitted through sexual behavior in children. As such, we propose a possible mode of transmission that is similar to that of *Enterobius vermicularis*. Although the prevalence of *E*. *vermicularis* was not investigated in this study, a prevalence of 7.8% has previously been reported in 2 primary schools in Tak Province [43]. Further study is required to test this hypothesis through simultaneous detection of *Entamoeba* cysts and *E*. *vermicularis* eggs in samples of dirt collected from under the fingernails. Indeed, cleanliness of the fingernails has been shown to have a significant effect on the prevalence of intestinal parasitic infections in school children [44].

In the present study, students who had siblings in the same school showed a significantly higher prevalence of *E*. *dispar* and *E*. *coli* infections than students who did not have siblings in the school, although the higher prevalence of *E*. *histolytica* was not significant, suggesting that transmission of *Entamoeba* between siblings occurred at home. Indeed, the fact that four of six sets of siblings with *E*. *histolytica* and *E*. *dispar* infections had identical genotypes at the D-A locus further supports the significance of this transmission route. The prevalence of genotype Ed5DA in Pri-3d and Pri-2d of school B may be due to familial transmission. Because there were siblings infected with *E*. *dispar* showing an identical genotype, Ed4DA, in Pri-5a and Pri-5b, it might be possible that the siblings transmitted the infection from home to two classrooms in the school. Another possibility is that one of the siblings was infected in a classroom of the school, carried the infection to home and transmitted it to siblings, and then transmission was extended to another classroom from home. There is also a possibility of transmission from school to home and vice versa [20]. It is interesting and important to follow the direction of transmission. For further analysis, the following questions are important and need to be answered: Are the children infected before they enter into the school? Are their families exposed to the parasite or infected? Were children infected in the school? Initial infection might occur at home; however, it is difficult to prove the direction of transmission and to ascertain how to transmit the infections between home and school in this study, because of the limitations of it being a cross sectional study. Further studies would be required for answering these questions.

By contrast, we previously reported different *E*. *dispar* genotypes in a couple from a family in Nepal [21]. Different *E*. *dispar* genotypes at another locus have also been observed in children and their relatives in Mexico [20]. There may be differences in behavior between adults and children that affect transmission. It is probable that contact with siblings is closer than that with the parents at home. Further analysis of family members is required to confirm transmission within families, as the possibility of transmission by contaminated drinking water and foods in the homes could not be excluded.

Outbreaks of amebiasis have also been reported in institutions for individuals with mental disabilities [45–49]. Abnormal behaviors such as pica and fecal play (coprophilia) are suggested to be factors that promote the transmission of *Entamoeba*. However, the teachers reported that no such behavior was observed in the students from the schools studied.

A high prevalence of *E*. *moshkovskii* infection has recently been reported in rural communities of many countries. A prevalence rate of 18.2% was reported in Yemen [50], 15.9% in South Africa [30], 21.1% in preschoolers in Bangladesh [51], 12.3% in Malaysia [52], 61.8% in patients in Australia [53], and 13% for suspected HIV or HIV-positive inpatients in Tanzania [54]. However, no *E*. *moshkovskii* infections were detected in this study or in our previous study in Nepal [21]. In the present study, we designed a new primer set for amplification of the *E*. *moshkovskii 18S* rRNA gene, which covered the sequences recently stored in DNA databases. These sequences were from *E*. *moshkovskii* isolates from human (KP722601-KP722605), snake (MN536488), cockroach (MN535795, MN535796, MN536492), beetle (MN536495), and various water sediments (MN536493, MN536494, MN536496-MN536501). The primer set was shown to effectively amplify the partial *18S* sequence of Laredo strain (AF149906) (S1 Fig). Although *E*. *moshkovskii* has previously been detected in clinical samples, its prevalence may be lower or may not be widely distributed in Thailand [31,32].

*E*. *nuttalli* and *E*. *chattoni* infections were also not detected. Macaques are the natural host of these parasites, and although we had previously reported *E*. *chattoni* infection in Nepal where macaques live in the same area as the study participants, few macaques are found in the studied area in Thailand [21]. Despite the presence of pigs that were kept near school B, *E*. *polecki* infection was not detected.

In conclusion, the mode of transmission of *E*. *histolytica* and *E*. *dispar* among school children on the Thai-Myanmar border region appears to be through direct or indirect person-to-person contact within classes, and it also seems to occur in siblings at home due to their more extensive contact. These findings suggest that specific measures are necessary to prevent transmission in both schools and at home.

## Supporting information

S1 Fig PCR amplification of the partial *18S* rRNA gene from *E*. *moshkovskii*. Genomic DNA isolated from *E*. *moshkovskii* Laredo strain was serially diluted and used as a template for PCR (lanes 1 to 7). Predicted 200-bp products were clearly detected from 1 pg of template DNA (lane 5). Lane 8, without template DNA; M, 100-bp DNA ladder.
(TIF)

S1 Table Chi-square test of the prevalence of the three *Entamoeba* species shown in Table 4. (DOCX)

S2 Table Chi-square test of the prevalence of genotype Eh5DA. (DOCX)

S3 Table Chi-square test of the prevalence of genotype Ed4DA. (DOCX)

S4 Table Chi-square test of the prevalence of genotype Ed7DA. (DOCX)

S5 Table Chi-square test of the prevalence of genotype Ed5DA. (DOCX)
