# Supplementary figures and images for: Analysis of D-A locus of tRNA-linked short tandem repeats reveals transmission of Entamoeba histolytica and E. dispar among students in the Thai-Myanmar border region of northwest Thailand
Source: PLoS Negl Trop Dis. 2021 Feb 18;15(2):e0009188. doi: 10.1371/journal.pntd.0009188 (PMC7924757; doi:10.1371/journal.pntd.0009188)

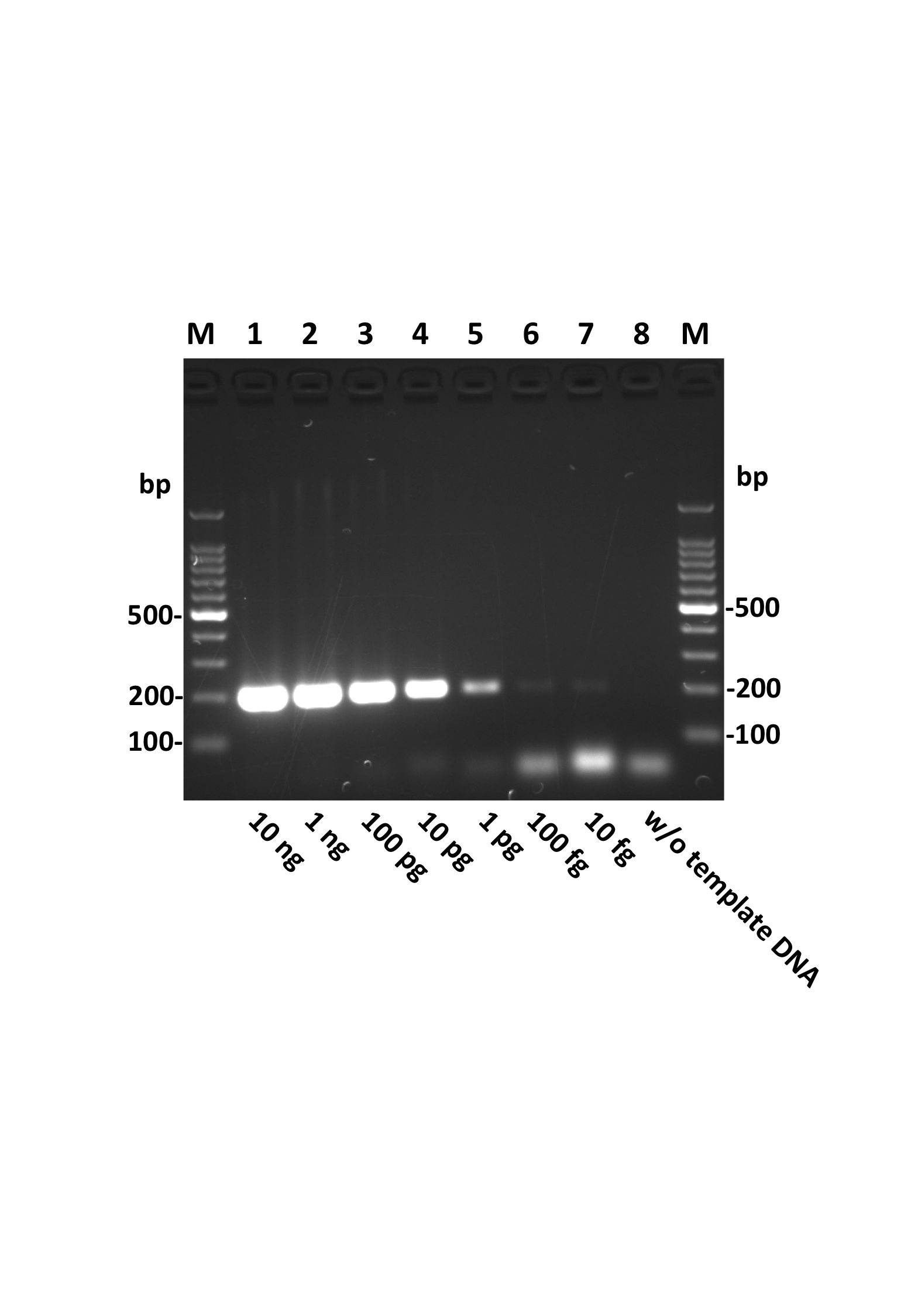

Supplement: S1 Fig — Genomic DNA isolated from E. moshkovskii Laredo strain was serially diluted and used as a template for PCR (lanes 1 to 7). Predicted 200-bp products were clearly detected from 1 pg of template DNA (lane 5). Lane 8, without template DNA; M, 100-bp DNA ladder. (TIF) [file pntd.0009188.s001.tif]
