# Supplementary material for: Analysis of D-A locus of tRNA-linked short tandem repeats reveals transmission of Entamoeba histolytica and E. dispar among students in the Thai-Myanmar border region of northwest Thailand
Source: PLoS Negl Trop Dis. 2021 Feb 18;15(2):e0009188. doi: 10.1371/journal.pntd.0009188 (PMC7924757; doi:10.1371/journal.pntd.0009188)
Supplement: S1 Table — (DOCX) [file pntd.0009188.s002.docx]

| School | *E. histolytica*  Positive (n) | *E. histolytica*  Negative (n) |  | Comparison | Chi-square | Odds ratio (95% CI) | *p* value |
| --- | --- | --- | --- | --- | --- | --- | --- |
| A | 20 | 788 |  | A vs B | 2.996 | 0.3469 (0.09901-1.215) | 0.0835 |
| B | 3 | 41 |  | B vs C | 3.483 | 3.412 (0.8705-13.37) | 0.0620 |
| C | 8 | 373 |  | A vs C | 0.1588 | 1.183 (0.5164-2.712) | 0.6903 |
|  | *E. dispar*  Positive (n) | *E. dispar*  Negative (n) |  |  |  |  |  |
| A | 39 | 769 |  | A vs B | 19.17 | 0.1972 (0.08860-0.4390) | <0.0001* |
| B | 9 | 35 |  | B vs C | 37.74 | 13.74 (4.822-39.14) | <0.0001* |
| C | 7 | 374 |  | A vs C | 6.222 | 2.710 (1.200-6.116) | 0.0126* |
|  | *E. coli*  Positive (n) | *E. coli*  Negative (n) |  |  |  |  |  |
| A | 189 | 619 |  | A vs B | 52.87 | 0.1145 (0.05781-0.2268) | <0.0001* |
| B | 32 | 12 |  | B vs C | 89.99 | 17.65 (8.531-36.53) | <0.0001* |
| C | 50 | 331 |  | A vs C | 17.00 | 2.021 (1.440-2.837) | <0.0001* |

S1 Table. Chi-square test of the prevalence of three *Entamoeba* species in Table 4.

*Statistically significant
