# Supplementary material for: Analysis of D-A locus of tRNA-linked short tandem repeats reveals transmission of Entamoeba histolytica and E. dispar among students in the Thai-Myanmar border region of northwest Thailand
Source: PLoS Negl Trop Dis. 2021 Feb 18;15(2):e0009188. doi: 10.1371/journal.pntd.0009188 (PMC7924757; doi:10.1371/journal.pntd.0009188)
Supplement: S2 Table — (DOCX) [file pntd.0009188.s003.docx]

| Group | School and Class | Eh5DA (n) | Other genotypes (n) | Comparison | Chi-square | Odds ratio (95% CI) | *p* value |
| --- | --- | --- | --- | --- | --- | --- | --- |
| 1 | B-Pri-1d | 2 | 0 | Group 1 vs Groups 2-4 | 7.879 | 35.00 (1.072-1143) | 0.0050* |
| 2 | B-Pri-3d | 1 | 0 | Group 2 vs Groups 1, 3, and 4 | 3.611 | 12.60 (0.3858-411.5) | 0.0574 |
| 3 | A | 0 | 5 | Groups 1 and 2 vs Group 3 | 8.000 | 77.00 (1.222-4854) | 0.0047* |
| 4 | C | 0 | 5 | Groups 1 and 2 vs Group 4 | 8.000 | 77.00 (1.222-4854) | 0.0047* |

S2 Table. Chi-square test of the prevalence of genotype Eh5DA.

*Statistically significant
