# Supplementary material for: Analysis of D-A locus of tRNA-linked short tandem repeats reveals transmission of Entamoeba histolytica and E. dispar among students in the Thai-Myanmar border region of northwest Thailand
Source: PLoS Negl Trop Dis. 2021 Feb 18;15(2):e0009188. doi: 10.1371/journal.pntd.0009188 (PMC7924757; doi:10.1371/journal.pntd.0009188)
Supplement: S3 Table — (DOCX) [file pntd.0009188.s004.docx]

| Group | School and Class | Ed4DA (n) | Other genotypes (n) |  | Comparison | Chi-square | Odds ratio (95% CI) | *p* value |
| --- | --- | --- | --- | --- | --- | --- | --- | --- |
| 1 | A-Pri-5a | 7 | 0 |  | Group 1 vs Groups 2-6 | 2.874 | 7.222 (0.3551-146.9) | 0.0900 |
| 2 | A-Pri-5b | 7 | 1 |  | Group 1 vs Groups 2-9 | 5.077 | 12.93 (0.6689-250.0) | 0.0242* |
| 3 | A-Pri-4a | 3 | 1 |  | Group 1 vs Groups 2-11 | 10.18 | 27.41 (1.458-515.5) | 0.0014* |
| 4 | A-Pri-6c | 2 | 0 |  | Group 2 vs Groups 1, and 3-9 | 2.599 | 5.500 (0.5857-51.65) | 0.1069 |
| 5 | A-Pri-1a | 1 | 1 |  | Group 2 vs Groups 1, and 3-11 | 7.152 | 12.50 (1.391-112.3) | 0.0075* |
| 6 | A-Pri-others | 0 | 3 |  | Groups 1 and 2 vs Groups 3-6 | 5.379 | 11.67 (1.112-122.5) | 0.0204* |
| 7 | A-Sec-3c | 1 | 0 |  | Groups 1 and 2 vs Groups 3-9 | 10.48 | 22.00 (2.343-206.6) | 0.0012* |
| 8 | A-Sec-others | 0 | 3 |  | Groups 1 and 2 vs Groups 3-11 | 21.10 | 50.00 (5.565-449.3) | <0.0001* |
| 9 | A-Kin | 0 | 3 |  | Groups 1-6 vs Groups 7-9 | 9.351 | 20.00 (1.994-200.6) | 0.0022* |
| 10 | B | 0 | 9 |  | Groups 1-9 vs Group 10 | 11.45 | 32.68 (1.747-611.2) | 0.0007* |
| 11 | C | 0 | 5 |  | Groups 1-9 vs Group 11 | 7.112 | 18.92 (0.9624-372.0) | 0.0077* |

S3 Table. Chi-square test of the prevalence of genotype Ed4DA.

*Statistically significant
