# Supplementary material for: Analysis of D-A locus of tRNA-linked short tandem repeats reveals transmission of Entamoeba histolytica and E. dispar among students in the Thai-Myanmar border region of northwest Thailand
Source: PLoS Negl Trop Dis. 2021 Feb 18;15(2):e0009188. doi: 10.1371/journal.pntd.0009188 (PMC7924757; doi:10.1371/journal.pntd.0009188)
Supplement: S4 Table — (DOCX) [file pntd.0009188.s005.docx]

| Group | School and Class | Ed7DA (n) | Other genotypes (n) |  | Comparison | Chi-square | Odds ratio (95% CI) | *p* value |
| --- | --- | --- | --- | --- | --- | --- | --- | --- |
| 1 | B-Pri-1d | 3 | 0 |  | Group 1 vs Group 2 | 9.000 | 91.00 (1.462-5662) | 0.0027* |
| 2 | B-Pri-others | 0 | 6 |  | Group 1 vs Groups 2-5 | 34.45 | 203.0 (6.900-5972) | <0.0001* |
| 3 | C-Sec-4b | 1 | 2 |  | Groups 1 and 2 vs Groups 3 and 4 | 0.2800 | 2.000 (0.1495-26.75) | 0.5967 |
| 4 | C-others | 0 | 2 |  | Groups 1 and 2 vs Group 5 | 11.85 | 36.08 (1.657-785.5) | 0.0006* |
| 5 | A | 0 | 33 |  |  |  |  |  |

S4 Table. Chi-square test of the prevalence of genotype Ed7DA.

*Statistically significant
