# Supplementary material for: Analysis of D-A locus of tRNA-linked short tandem repeats reveals transmission of Entamoeba histolytica and E. dispar among students in the Thai-Myanmar border region of northwest Thailand
Source: PLoS Negl Trop Dis. 2021 Feb 18;15(2):e0009188. doi: 10.1371/journal.pntd.0009188 (PMC7924757; doi:10.1371/journal.pntd.0009188)
Supplement: S5 Table — (DOCX) [file pntd.0009188.s006.docx]

| Group | School and Class | Ed5DA (n) | Other genotypes (n) |  | Comparison | Chi-square | Odds ratio (95% CI) | *p* value |
| --- | --- | --- | --- | --- | --- | --- | --- | --- |
| 1 | B-Pri-3d | 3 | 0 |  | Group 1 vs Groups 2 and 3 | 3.600 | 12.60 (0.4451-356.7) | 0.0578 |
| 2 | B-Pri-2d | 2 | 0 |  | Group 1 vs Group 3 | 7.000 | 63.00 (0.9809-4046) | 0.0082* |
| 3 | B-Pri-others | 0 | 4 |  | Group 1 vs Groups 2-6 | 21.90 | 83.00 (3.527-1953) | <0.0001* |
| 4 | A-Pri-4b | 1 | 0 |  | Group 2 vs Group 3 | 6.000 | 45.00 (0.6649-3046) | 0.0143* |
| 5 | A-others | 0 | 32 |  | Groups 1 and 2 vs Group 3 | 9.000 | 99.00 (1.618-6059) | 0.0027* |
| 6 | C | 0 | 5 |  | Groups 1-3 vs Groups 4 and 5 | 15.93 | 40.00 (3.680-434.8) | <0.0001* |
|  |  |  |  |  | Groups 1-3 vs Group 6 | 4.321 | 13.44 (0.5747-314.5) | 0.0376* |

S5 Table. Chi-square test of the prevalence of genotype Ed5DA.

*Statistically significant
